# Filling ability and blooming artifact expression in long-oval root canals: influence of CBCT device, endodontic sealer, and BAR filter

**DOI:** 10.1007/s11282-026-00905-x

**Published:** 2026-02-21

**Authors:** Gustavo Ovigli, Airton Oliveira Santos-Junior, Karina Ines Medina Carita Tavares, Juliane Maria Guerreiro-Tanomaru, Mario Tanomaru-Filho, Fernanda Ferrari Esteves Torres

**Affiliations:** Araraquara Dental School, Department of Restorative Dentistry, São Paulo State University, Araraquara, SP Brazil

**Keywords:** Artifacts, Cone beam computed tomography, Dental materials, Diagnostic imaging, Endodontics, Micro-computed tomography

## Abstract

**Objectives:**

To compare the filling ability of long oval root canals with epoxy resin–based and bioceramic sealers, and to assess the influence of two CBCT devices and the blooming artifact reduction (BAR) filter (e-Vol DX Software) on dimensional analysis of root canal fillings, with micro-CT as reference.

**Methods:**

Mandibular incisors were prepared and filled with AH Plus Jet or Bio-C Sealer, then scanned with VeraView X800 and OP300 Maxio CBCT devices, with micro-CT as the reference. Micro-CT images were analyzed to determine the filling ability of each sealer. Three blinded examiners assessed CBCT images, and dimensional analyses compared measurements before and after BAR filter application. Unpaired t-test, ICC, and ANOVA/Tukey were applied (α = 5%).

**Results:**

Both sealers showed high filling ability (*p* > 0.05). Intra- and inter-examiner agreement demonstrated excellent reproducibility (*p* < 0.001). Both CBCT devices overestimated canal filling, particularly VeraView X800 without the filter. Application of the BAR filter reduced blooming artifacts in VeraView X800 scans, enhancing dimensional assessment of the root canal in the apical third for AH Plus Jet and across all thirds for Bio-C Sealer (*p* < 0.05).

**Conclusions:**

Both sealers exhibited high and comparable filling ability. The BAR filter reduced blooming artifacts in VeraView X800 images, with more consistent effect observed for Bio-C Sealer.

## Introduction

 Three-dimensional root canal obturation is essential for endodontic treatment success, as it ensures an effective seal and prevents reinfection [1]. Both the endodontic sealer and root canal morphology play key roles in this outcome. Bio-C Sealer (Angelus Indústria de Produtos Odontológicos, Londrina, PR, Brazil) is a bioceramic material that demonstrates biocompatibility and bioactivity [2], whereas AH Plus (Dentsply DeTrey GmbH, Konstanz, BW, Germany), an epoxy resin–based sealer, remains the gold standard due to its physicochemical properties [3]. Long-oval root canals, characterized by wide buccolingual dimensions, pose challenges for effective instrumentation and filling [4]. Mandibular incisors are representative of this morphology [5], allowing extrapolation of findings to other teeth with similar canal configurations [6].

Although previous studies have compared the filling ability of Bio-C Sealer and AH Plus [7, 8], no investigation has yet addressed their performance in filling long-oval root canals of mandibular incisors using the single-cone technique. This issue has direct clinical relevance, as filling homogeneity is strongly correlated with periapical lesion healing outcomes [9].

In clinical practice, root canal fillings are commonly assessed using periapical radiography, which provides only a two-dimensional view of a three-dimensional structure [10]. Cone-beam computed tomography (CBCT) enables three-dimensional evaluation of filling quality, including voids that may compromise treatment success [11]. Careful analysis of root fillings is critical in cases of endodontic treatment failure, as it supports retreatment planning and helps avoid unnecessary tooth extractions [10, 12, 13]. However, CBCT accuracy may be limited by artifacts generated by high-density filling materials, leading to misinterpretation or diagnostic errors [14–19].

Blooming is a frequent CBCT artifact caused by the high density of gutta-percha and endodontic sealers, resulting in volumetric distortion and unreliable image magnification [17, 20]. Its expression varies according to the radiopacity of the sealer, but evidences regarding bioceramic sealers remains limited, particularly with respect to quantitative data on volumetric distortion [16]. Artifact formation is also influenced by technical acquisition parameters such as field of view (FOV), voxel size, milliamperage (mA), and kilovoltage (kV) [15, 21]. Thus, comparing different CBCT devices is clinically relevant for identifying the most effective system in tasks such as retreatment planning [22].

Aiming to improve CBCT image quality and diagnostic accuracy, the e-Vol DX software (CDT Software, Bauru, SP, Brazil) has been investigated, particularly for its Blooming Artifact Reduction (BAR) filter, designed to optimize brightness and contrast [23]. However, its effectiveness remains controversial. While some studies report that the BAR filter does not successfully reduce blooming artifacts [21, 24], others highlight its potential to enhance CBCT image quality [25, 26]. A recent study emphasized the importance of evaluating new CBCT tools, including protocols and filters, and stressed the need for research in different fields to understand their potential benefits for specific specialties [27]. The BAR filter has been shown to improve dimensional analysis of implants by reducing artifacts [26], but its effectiveness in optimizing image quality for root canal fillings remains unexplored.

Therefore, this study aimed to compare the filling ability of long-oval root canals using an epoxy resin–based sealer (AH Plus Jet) and a bioceramic sealer (Bio-C Sealer) through micro-CT analysis, in addition to blooming artifacts in CBCT images with and without BAR filter application, using micro-CT as reference. The null hypotheses were: (1) AH Plus Jet and Bio-C Sealer would show similar filling ability in long-oval root canals; (2) the OP300 Maxio and VeraView X800 CBCT devices would show comparable distortion caused by blooming artifacts; (3) the BAR filter would not reduce blooming artifacts during CBCT image analysis.

## Materials and methods

### Sample size determination

Sample size was calculated using the G*Power software (version 3.1.7; Heinrich-Heine-Universität, Düsseldorf, Germany). Based on the analysis, ten teeth per group was determined to achieve statistical significance, with an alpha level of 0.05, a beta power of 0.80, and an effect size of 1.39 [16].

### Selection of teeth

**Fig. 1:**
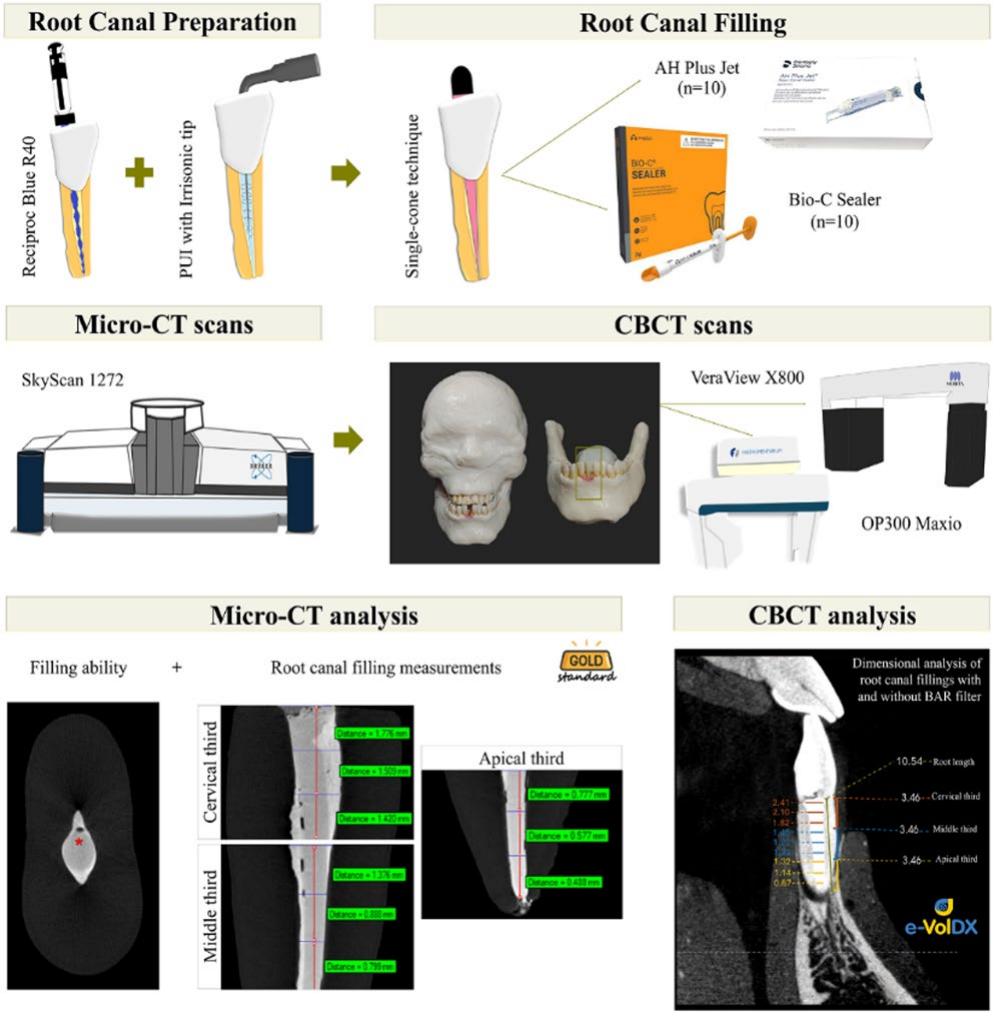
Flowchart illustrating the methodological approach adopted in the study

Under the ethical standards laid down in the 1964 Declaration of Helsinki and its later amendments, this study was approved by the institutional ethics committee (CAAE: 41916720.7.0000.5416 and 84582824.0.0000.5416). Twenty extracted human mandibular incisors with root canals classified as Vertucci’s type I [28] were selected. Inclusion criteria included complete apical formation, absence of root fractures, calcifications, previous endodontic treatment, or internal/external resorption. All root canals presented a long-oval cross-section, as determined by digital radiography (Kodak RVG 6100; Digital Radiography System, Marne-la-Vallée, France) and micro-CT scans (SkyScan 1272; Bruker MicroCT, Kontich, Belgium). The criteria for classifying the canals as long-oval were a buccolingual diameter 2 to 4 times larger than the mesiodistal diameter [29] at 9 mm from the root apex [5]. Measurements were performed using CTAn software (v. 1.15.4.0; Bruker MicroCT). A flowchart is presented in Fig. 1, outlining the sequential methodological steps followed in this investigation.

### Root Canal Preparation

Coronal access was obtained using a #1012 diamond bur (Dentsply Sirona, Ballaigues, Switzerland). The root canals were explored with #10 C+ files (Dentsply Sirona), and the working length (WL) was determined by subtracting 1.0 mm from the point at which the file tip was visible in the apical foramen. Root canal preparation was performed with the Reciproc Blue R40 instrument (VDW GmbH, Munich, Germany) up to the WL, operated using the X-Smart Plus motor (Dentsply Sirona). Irrigation was performed with 6.0 mL of 2.5% sodium hypochlorite (NaOCl) (Ciclo Farma, Serrana, SP, Brazil) after each instrument, in 2 mL portions into the cervical, middle, and apical thirds of the root canal using a 30-G Navitip needle (Ultradent Products). The needle was positioned 2 mm short of the WL with back-and-forth movements under flow and aspiration. The final irrigation protocol consisted of 5 mL of 2.5% NaOCl, followed by 2 mL of 17% EDTA (Biodynamics, Ibiporã, PR, Brazil), and 5 mL of saline solution, all activated by passive ultrasonic irrigation (PUI) with an Irrisonic tip (Helse Ultrasonic, Santa Rosa de Viterbo, SP, Brazil). Finally, the canals were aspirated and dried with absorbent paper points (Dentsply Sirona).

### Root Canal filling

All root canals were filled using the single-cone technique with AH Plus Jet or Bio-C Sealer (*n* = 10 per group). R40 gutta-percha cones (VDW GmbH) were selected with a profilometer device (Profile Projector Nikon model 6 C-2; Nikon, Tokyo, Japan). Digital radiographs were taken to confirm the adaptation of the gutta-percha cones at the WL. For root canals filled with AH Plus Jet, 1 g of sealer was prepared by dispensing equal lengths of pastes A and B, then mixed for 30 s with a #24 metal spatula (Duflex, Juiz de Fora, MG, Brazil) on a glass slab (Golgran, São Caetano do Sul, Brazil) until a homogeneous consistency was achieved [30]. The sealer was applied using a #40 Lentulo spiral (Dentsply Sirona) connected to a low-speed motor (Micromotor N270 with contra-angle, Dabi-Atlante, Ribeirão Preto, SP, Brazil), positioned 2 mm short of the WL [7]. Bio-C Sealer was injected into the root canals using syringes and plastic needles positioned 4 mm short of the WL according to the manufacturer’s instructions. The syringe plunger was pressed lightly until the sealer flowed back into the cervical third, indicating complete filling of the root canals [7]. The R40 gutta-percha cones were coated with the respective sealers and inserted to the WL. Excess gutta-percha was removed from the cervical third and compacted vertically with a Paiva #2 plugger (Golgran). Final digital radiographs were obtained in both mesiodistal and buccolingual directions to confirm the absence of voids and the uniformity of fillings. The root canals were restored with Coltosol (Vigodent, Rio de Janeiro, RJ, Brazil), and the specimens were stored at 37 °C and 95% relative humidity for one week to allow complete sealer setting [31].

### Micro-CT acquisition

To obtain the gold-standard reference for comparisons with CBCT scans, the teeth were individually scanned using a high-resolution micro-CT device (SkyScan 1272; Bruker MicroCT). The scanning parameters were: voxel size of 9 μm, 1-mm aluminum filter, exposure time of 87 ms, 180° rotation, rotation step of 0.5°, 80 kV and 300 µA.

### CBCT acquisition

For CBCT imaging, an anthropomorphic model consisting of a skull and a fully dentate mandible was used. The phantom was coated with Mix-D, a material designed to simulate X-ray attenuation similar to human soft tissues, and also served to create a tongue model [31]. The left mandibular central incisor of the dry mandible was carefully removed, and the prepared teeth were individually positioned in the empty sockets. Two CBCT devices were used: VeraView X800 (J Morita, Tokyo, Japan) and OP300 Maxio (Instrumentarium Dental, Tuusula, Finland). For the VeraView X800, images were acquired with a voxel size of 0.08 mm, exposure settings of 70 kV and 5 mA, and a field of view (FOV) of 40 × 40 mm. For the OP300 Maxio, acquisition was performed with a voxel size of 0.08 mm, 90 kV, 8 mA, and an FOV of 50 × 50 mm.

### Micro-CT image analysis

The micro-CT images were reconstructed using NRecon software (v1.6.3, Bruker MicroCT) with specific artifact correction adjustments, including beam hardening, ring and smoothing corrections, according to the sealer used (AH Plus Jet or Bio-C Sealer). For quantitative analysis, a region of interest (ROI) corresponding to the root canal space was defined. From this region, the total volume (mm³) of the filling material, as well as the percentage of filled areas (%) and voids (%), were calculated. Global image segmentation was performed using the automatic thresholding plug-in of CTAn software, with manual adjustments when necessary.

The micro-CT images were also used to establish the diameter of the root canal filling material and to detect possible measurement distortions for canals filled with either AH Plus Jet or Bio-C Sealer. The analysis was performed in the sagittal plane using CTAn software to assess the buccolingual dimensions of the filling material at the cervical, middle, and apical thirds of the root canal. For each specimen, the sagittal reconstruction plane was carefully adjusted to pass through the center of the root canal filling and aligned with the long axis of the root. The same anatomical references and plane orientation were used for all specimens and imaging modalities. These data served as the gold standard for comparisons with CBCT-derived measurements, obtained with and without the BAR filter. The examiner responsible for the micro-CT measurements was not involved in evaluations of the CBCT images.

### CBCT image analysis

CBCT images were analyzed using e-Vol DX (CDT Software, Bauru, SP, Brazil) by three examiners, who were instructed to perform detailed measurements across the cervical, middle, and apical thirds of root canals filled with AH Plus Jet or Bio-C Sealer. A calibration process was performed using random images, in which the examiners were shown how to analyze the images under the same conditions. Subsequently, each examiner individually analyzed four random images, including variations of CBCT devices, root canal sealers and application or absence of the BAR filter. The images used in the calibration process were not included in the final evaluation. The intraclass correlation coefficient (ICC) indicated good agreement between the examiners.

**Fig. 2:**
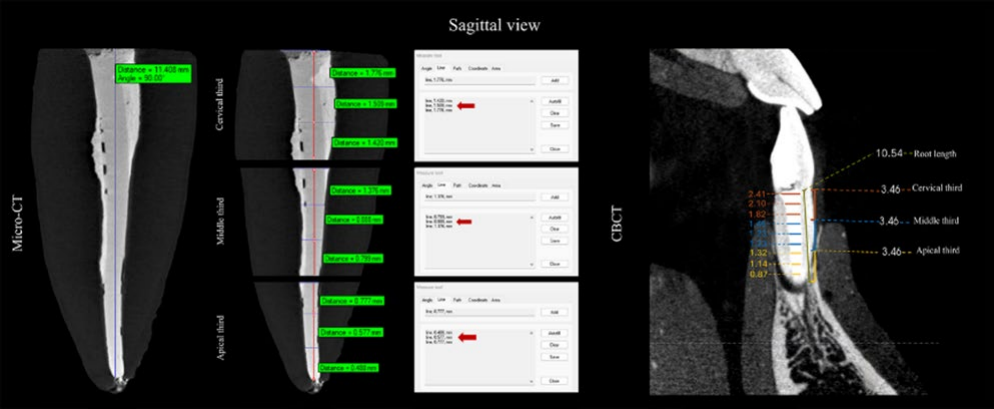
Sagittal reconstructions of a mandibular central incisor from the sample, illustrating buccolingual dimensional measurements of the filling material. Micro-CT images (left) served as the reference standard, while CBCT images (right) were analyzed to detect dimensional distortions in the cervical, middle, and apical thirds of the root canal, with and without application of the BAR filter

Once the examiners were familiarized with the measurement parameters and calibrated, the CBCT images were analyzed. The diameter of the root canal fillings was measured on sagittal reconstructions obtained from both CBCT devices, under two conditions: with and without the BAR filter. Sagittal reconstruction planes were defined using the same criteria adopted for the micro-CT analysis, being aligned with the long axis of the root canal and positioned to pass through the center of the filling material, thus ensuring that measurements were obtained from corresponding anatomical locations across CBCT and micro-CT images. To standardize the evaluation, each root was divided into three segments (cervical, middle, and apical) based on the location of the filling material. The measurements across the three segments were performed in triplicate. This approach ensured that all examiners assessed the root canals in a standardized manner [32]. All images were analyzed on a 25-inch color LED monitor with a resolution of 2560 × 1080 pixels, in a low-light and quiet room. To minimize visual fatigue, a maximum of five CBCT volumes were evaluated per day, with at least 24 h between evaluation sessions. To assess intra-examiner reproducibility, 30% of the sample was randomly selected and reanalyzed after a 30-day interval following the initial evaluation. Dimensional measurements on CBCT and micro-CT images were performed as illustrated in Fig. 2.

### Statistical analysis

Statistical analyses were performed using GraphPad Prism 9 (GraphPad Software. San Diego, CA, USA) and SPSS software (v24.0, IBM Corp., Armonk, NY, USA). The intraclass correlation coefficient (ICC) was calculated to assess intra-examiner reproducibility and inter-examiner reliability for CBCT analysis. Data normality was verified using the Shapiro–Wilk test. An unpaired t-test was used to compare the filling ability of the sealers based on micro-CT measurements. Repeated measures ANOVA followed by Tukey’s post hoc test was applied for multiple comparisons between the reference measurements (micro-CT) and those obtained from CBCT images with and without the BAR filter. A significance level of 5% was adopted for all analyses.

## Results

**Fig. 3:**
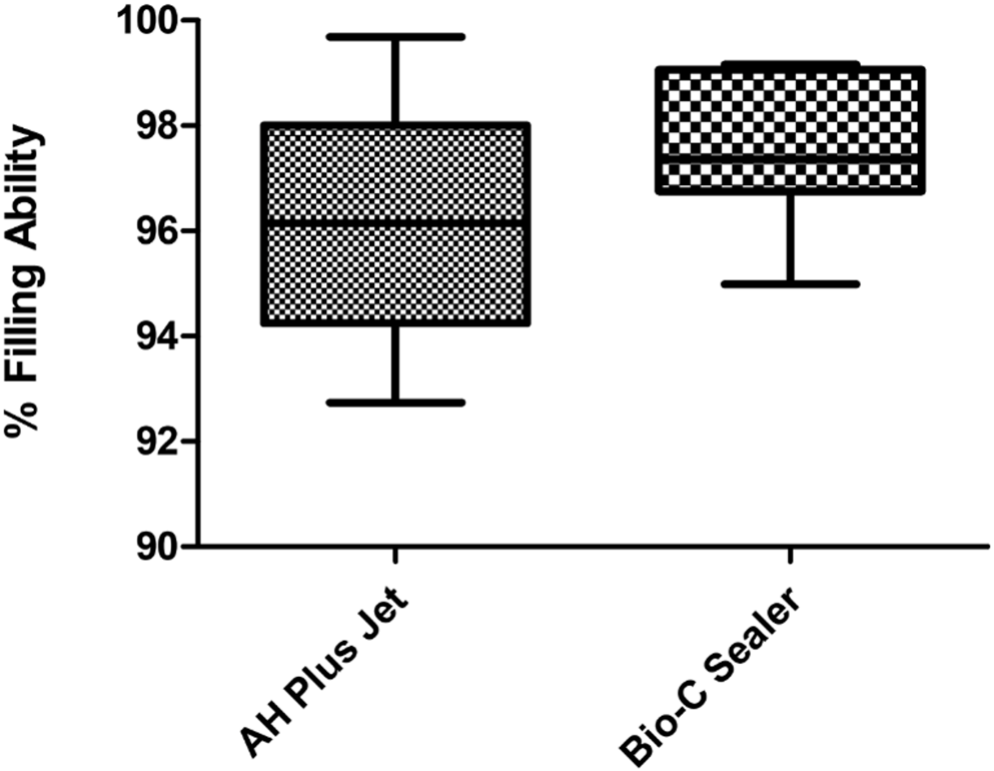
Boxplot showing the percentage (%) of filling ability for AH Plus Jet and Bio-C Sealer. Statistical analysis revealed no significant difference between the sealers (*p* > 0.05)

**Fig. 4:**
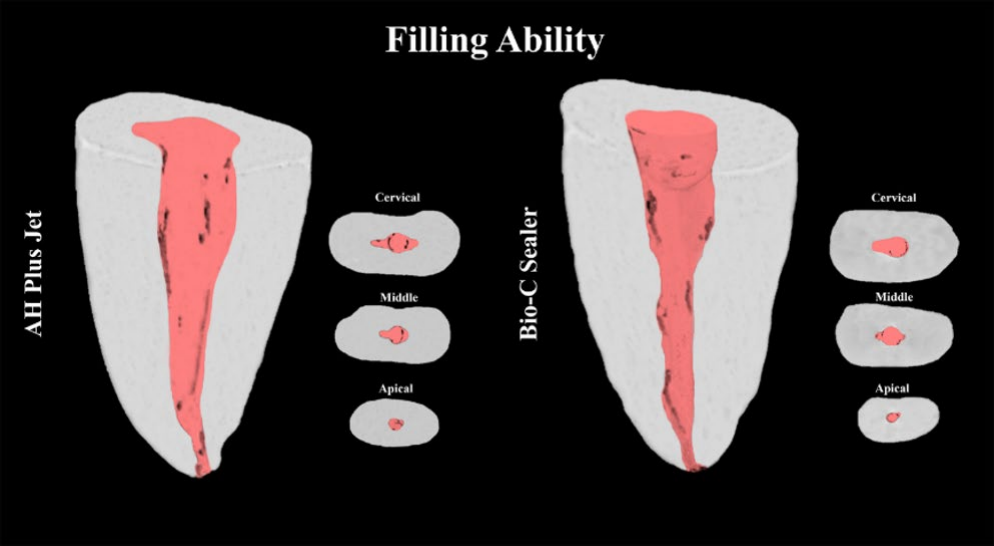
Three-dimensional and cross-sectional transparent views from micro-CT images illustrating the filling ability of AH Plus Jet and Bio-C Sealer. Reconstructions are displayed for the complete root canal and separately for the cervical, middle, and apical thirds. The filling material is shown in pink, and black areas represent voids within the filling

Figures 3 and 4 illustrate the filling ability of AH Plus Jet and Bio-C Sealer, when analyzed in micro-CT. Both sealers filled more than 90% of the root canal volume, with no statistically significant difference between them (*p* > 0.05).

Intra-examiner agreement in the CBCT image analysis demonstrated excellent reproducibility, with ICC values ranging from 0.930 to 0.986 (*p* < 0.001). These results indicate high reliability across repeated measurements. Table 1 presents the inter-examiner reliability among the three examiners, which was also classified as excellent, with ICC values ranging from 0.879 to 0.963 (*p* < 0.001).

**Table 1:** Mean (± standard deviation) (mm) of the measurements performed by each examiner and the reliability between repeated measurements

Interexaminer agreement	Root canal filling measurement
Examiner 1	1.29 (± 0.45)
Examiner 2	1.35 (± 0.45)
Examiner 3	1.49 (± 0.46)
ICC	0.937
CI (95%)	0.879–0.963
*P* Value	< 0.001

*ICC* intraclass correlation coefficient,* CI* confidence interval

**Fig. 5:**
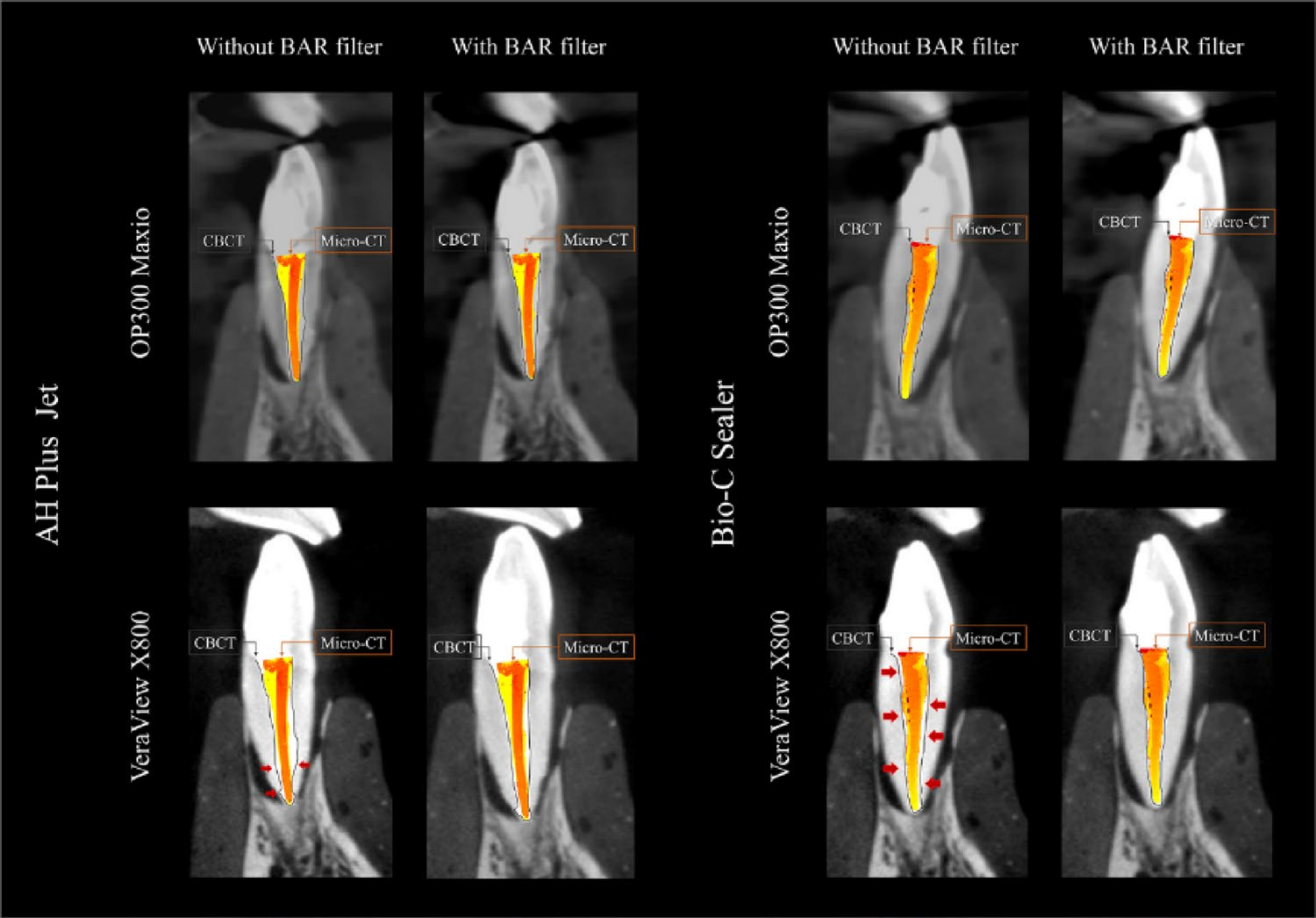
Sagittal CBCT reconstructions (OP300 Maxio and VeraView X800) of mandibular central incisors filled with AH Plus Jet and Bio-C Sealer, displayed without and with application of the BAR filter. The intracanal material segmented from micro-CT (gutta-percha in orange and sealer in yellow), serving as the reference standard, is superimposed into the CBCT images. Grey outlines denote CBCT-derived contours of the filling, highlighting dimensional overestimation caused by blooming artifacts; red arrows indicate the most affected regions

Table 2 shows the measurements of root canal filling from micro-CT (reference) and CBCT (OP300 Maxio and VeraView X800) images, with and without the application of the BAR filter. Both CBCT devices overestimated root canal filling compared with the micro-CT. Veraview X800 (without BAR filter) caused more pronounced distortions, mainly in the apical third, indicating greater susceptibility to the production of blooming artifacts and greater benefit in the application of the BAR filter. For AH Plus Jet, the BAR filter had a significant effect only in the apical third with the VeraView X800 (*p* < 0.05). For Bio-C Sealer, the BAR filter influenced the VeraView X800 measurements across all thirds evaluated (*p* < 0.05) (Fig. 5).

**Table 2:** Mean (± standard deviation) (mm) of dimensional analyses of different root Canal thirds filled with AH plus jet and Bio-C Sealer. Analyses were performed on images acquired using OP300 Maxio and VeraView X800 CBCT devices, with and without the BAR filter. Micro-CT was used as the reference

	Micro-CT	OP300 Maxio without BAR filter	OP300 Maxio with BAR filter	VeraView X800 without BAR filter	VeraView X800 with BAR filter
Cervical third					
AH Plus Jet Bio-C Sealer	1.38 (± 0.32)^a^ 1.16 (± 0.30)^a^	1.90 (± 0.28)^b^ 1.70 (± 0.24)^bc^	1.85 (± 0.26)^b^ 1.62 (± 0.25)^bc^	2.11 (± 0.39)^b^ 1.95 (± 0.32)^c^	1.83 (± 0.43)^b^ 1.48 (± 0.33)^ab^
Middle third					
AH Plus Jet Bio-C Sealer	1.01 (± 0.28)^a^ 0.90 (± 0.21)^a^	1.47 (± 0.20)^b^ 1.22 (± 0.16)^b^	1.43 (± 0.20)^b^ 1.19 (± 0.16)^b^	1.63 (± 0.22)^b^ 1.48 (± 0.24)^c^	1.44 (± 0.25)^b^ 1.25 (± 0.20)^b^
Apical third					
AH Plus Jet Bio-C Sealer	0.52 (± 0.15)^a^ 0.53 (± 0.09)^a^	0.95 (± 0.07)^b^ 0.89 (± 0.10)^b^	0.93 (± 0.09)^b^ 0.84 (± 0.07)^b^	1.11 (± 0.17)^c^ 1.07 (± 0.12)^c^	0.94 (± 0.13)^b^ 0.90 (± 0.11)^b^

Different superscript letters indicate statistically significant differences among imaging methods (Micro-CT and CBCT devices, with and without the BAR filter) within the same root canal third and sealer (repeated measures ANOVA followed by Tukey’s post hoc test; *p* < 0.05). Values sharing at least one common letter are not statistically different

## Discussion

An adequate obturation combined with a hermetic seal is one of the main objectives of root canal therapy [33]. In laboratory investigations, micro-CT is recognized as the gold standard for assessing endodontic procedures, including the filling ability of root canal sealers [8]. The aim of the present study was to evaluate the filling ability of AH Plus Jet (epoxy resin-based) and Bio-C Sealer (bioceramic) in long-oval canals using micro-CT images. Additionally, volumetric distortion on CBCT images obtained with OP300 Maxio and VeraView X800 devices, with and without the application of the blooming artifact reduction (BAR) filter, was assessed, with micro-CT serving as the reference. Micro-CT analysis showed that both sealers achieved more than 90% of filling, with no statistically significant difference, supporting acceptance of the first null hypothesis. These results are consistent with previous reports evaluating these sealers in other anatomical configurations of root canals [2, 8]. Although both sealers performed adequately, the literature indicates that thermoplastic techniques may further improve filling quality in complex canal anatomies compared with the single-cone technique used in this study [4].

In cases where previous endodontic treatment is suspected to have failed, accurate evaluation of root canal filling is essential to guide retreatment planning and to avoid unnecessary procedures, such as tooth extraction [13]. Given the limitations of two-dimensional radiography, CBCT has emerged as a valuable resource for the diagnosis, treatment planning, and follow-up of endodontic procedures [17]. In our study, both CBCT devices overestimated filling volume compared with micro-CT, confirming that high-density intracanal materials (i.e., endodontic sealer and gutta-percha) generate blooming artifacts that directly affect volumetric measurements on CBCT images [15]. For this reason, micro-CT was used as the reference standard, given its superior spatial resolution obtained with smaller voxel sizes and longer exposure times, which enable more accurate dimensional analysis of the filling material and minimize the influence of blooming artifacts [17]. Nevertheless, artifact-related distortions remain a recurrent limitation of CBCT imaging in endodontics [26]. Therefore, this investigation evaluated the Blooming Artifact Reduction (BAR) filter to determine its influence on dimensional analysis, specifically whether its application could attenuate blooming artifacts and improve the reliability of measurements across different CBCT devices and endodontic sealers.

The second null hypothesis of the present study assumed that the OP300 Maxio and VeraView X800 CBCT devices would produce similar distortion of the filling material from blooming artifacts. This hypothesis was rejected, as the VeraView X800 consistently produced greater distortion than the OP300 Maxio. In this investigation, the VeraView X800 was operated at 70 kV and 5 mA, whereas the OP300 Maxio was operated at 90 kV and 8 mA. Since both CBCT devices were used with the same voxel size (0.08 mm³), the difference may be attributed to other acquisition parameters. Previous studies have shown that lower kilovoltage peaks are associated with increased blooming artifacts [15, 16]. Tube current may also have influenced the results, as higher kV and mA settings are typically applied in tasks that require greater image detail, such as detecting fine anatomical structures or when artifacts from high-density materials are expected [34]. Our findings are consistent with a recent study [35] that compared different CBCT acquisition protocols for detecting the second mesiobuccal canal in maxillary molars under artifact conditions. The authors reported that, for the VeraView X800, the highest image quality was achieved with the FOV 80 × 40 and voxel size of 0.125 mm³, whereas the FOV 40 × 40 and voxel size of 0.080 mm³ used in the present investigation resulted in poorer image quality and reduced diagnostic reliability. In contrast, the OP300 Maxio showed better diagnostic performance when operated with the FOV 50 × 50 and voxel size of 0.085 mm³, which was also applied in this study. Taken together, these results indicate that both the CBCT devices and the selected acquisition protocol play a decisive role in determining image quality and artifact expression [36].

The third null hypothesis of the current investigation, which stated that the application of the BAR filter would not reduce blooming artifacts during CBCT image analysis, was rejected. The results of this study demonstrated that the BAR filter improved image quality for the VeraView X800 unit, particularly in samples filled with Bio-C Sealer. Similarly, evidence from the literature reports comparable outcomes. A recent study [37] investigated the accuracy of CBCT images for measuring residual dentin thickness in teeth filled with radiodense intracanal materials when the BAR filter was applied. In that study, CBCT images were acquired with the CS 9600 unit (Carestream Dental LLC, Atlanta, GA, USA), and the BAR filter improved measurement accuracy by reducing blooming artifacts in the group with gutta-percha cones and Endofill sealer (Dentsply Maillefer), specifically in the cervical third. The agreement between the current investigation and that study may be explained by the comparable radiopacity of Bio-C Sealer and Endofill, as both exhibit values close to 5 mm aluminum (mm Al) [38, 39]. This similarity makes them susceptible to artifacts of comparable expression and, therefore, to a similar reduction when the BAR filter is applied.

Based on their lower radiopacity, bioceramic sealers would be expected to produce fewer blooming artifacts compared with epoxy resin–based sealers such as AH Plus [40]. However, our results showed that the BAR filter was effective for AH Plus only in the apical third, while its effect was evident across all thirds for Bio-C Sealer. The BAR filter was specifically developed to reduce blooming artifacts by minimizing volumetric distortion. It operates by optimizing brightness and contrast through the maximum dynamic range of DICOM files, thereby improving visualization in areas affected by high-density materials [41]. Nevertheless, the filter does not eliminate hyperdense or hypodense streaks and bands produced by the beam hardening phenomenon during CBCT acquisition. Such artifacts continue to compromise image interpretation in regions close to high-density structures [24]. Consequently, the present findings suggest that the BAR filter is more effective when the contrast between dentin and filling material is not excessively high. The superior effect found with Bio-C Sealer can likely be attributed to its intermediate density, permitting the filter to act more efficiently than in situations of marked radiopacity disparity, as observed with AH Plus.

Corroborating our findings, a previous study assessed the performance of a metal artifact reduction (MAR) algorithm in different CBCT units and reported distinct outcomes depending on the specific device used [20]. Specifically, the Picasso Trio unit (E-Woo Technology Co., Ltd./Vatech, Giheung-gu, Korea; 2010) underestimated gutta-percha when MAR was activated, whereas the OP300 unit consistently overestimated it, regardless of MAR activation. These results reinforce that, beyond the density of the filling material, the CBCT unit itself plays a decisive role in image accuracy. The authors also emphasized that, since artifacts may cause both overestimation and underestimation of filling quality, the parameters employed to assess endodontic treatment should be interpreted cautiously to reduce the likelihood of false-positive and false-negative diagnoses [20].

Several efforts were made to simulate clinical conditions in this study, including the use of an anthropomorphic model with a dry human skull and a fully dentate mandible, both covered with Mix-D material to reproduce the X-ray attenuation properties of soft tissues. In addition, a tongue model was crafted and positioned anatomically within the setup [31]. Nevertheless, some limitations should be acknowledged. Motion artifacts, which are common in clinical CBCT imaging and may compromise image quality [31], were not reproduced in our study. Moreover, the use of extracted teeth, although necessary for standardization, does not fully replicate the biological conditions of a clinical scenario. Only two CBCT systems, each operating under a single acquisition protocol, were evaluated, and the study was limited to two sealers and one obturation technique, while numerous alternatives are currently available in endodontic practice. Therefore, the results should be interpreted with caution, and further studies employing different diagnostic tasks, CBCT units, sealers, and obturation techniques are warranted to complement our findings.

## Conclusions

AH Plus Jet and Bio-C Sealer exhibited a high and similar filling ability in long-oval root canals. The dimensional analysis of both CBCT devices (OP300 Maxio and VeraView X800) overestimated canal filling measurements compared to micro-CT, with the VeraView X800 showing more pronounced distortions. The BAR filter enhanced the measurement accuracy of filled root canals only in the VeraView X800 group within the apical root third for AH Plus Jet and in the Bio-C Sealer group within all root thirds.

## Data Availability

The datasets generated and analyzed during the current study are available from the corresponding author on reasonable request.
